# Endoscopic Retrograde Cholangiopancreatography-Related Procedures for the Differential Diagnosis of Isolated Immunoglobin G4-Related Sclerosing Cholangitis and Perihilar Cholangiocarcinoma

**DOI:** 10.3390/diagnostics14151621

**Published:** 2024-07-26

**Authors:** Masaru Furukawa, Yasutaka Ishii, Yumiko Tatsukawa, Shinya Nakamura, Juri Ikemoto, Sayaka Miyamoto, Kazuki Nakamura, Yumiko Yamashita, Noriaki Iijima, Yasuhiro Okuda, Risa Nomura, Koji Arihiro, Keiji Hanada, Shiro Oka

**Affiliations:** 1 Department of Gastroenterology, Graduate School of Biomedical and Health Sciences, Hiroshima University, Hiroshima 734-8551, Japan; mfurukawa@hiroshima-u.ac.jp (M.F.);; 2 Department of Anatomical Pathology, Hiroshima University Hospital, Hiroshima 734-0037, Japan; arihiro@hiroshima-u.ac.jp; 3 Department of Gastroenterology, Onomichi General Hospital, Hiroshima 722-0018, Japan

**Keywords:** isolated IgG4-related sclerosing cholangitis, perihilar cholangiocarcinoma, endoscopic retrograde cholangiopancreatography, intraductal ultrasonography, peroral cholangioscopy

## Abstract

Background/Purpose: Differential diagnosis of isolated immunoglobin (Ig)G4-related sclerosing cholangitis (IgG4-SC) and cholangiocarcinoma is challenging. We aimed to clarify the role of endoscopic retrograde cholangiography (ERCP)-related procedures in the differential diagnosis of isolated IgG4-SC and perihilar cholangiocarcinoma (PHCC). Methods: Seven patients with hilar-type isolated IgG4-SC diagnosed at Hiroshima University Hospital and sixty-five patients with surgically resected invasive PHCC were enrolled, and the diagnostic yields of intraductal ultrasonography (IDUS), peroral cholangioscopy (POCS), and pathological examinations were determined. Results: In six of seven (86%) patients with isolated IgG4-SC, the stricture was in the perihilar bile duct. IDUS showed that symmetrical wall thickening (40% vs. 5%, *p* = 0.04), homogeneous internal echo (80% vs. 5%, *p* < 0.001), and smooth outer margins (80% vs. 6%, *p* < 0.001) were more frequent in isolated IgG4-SC than in PHCC. POCS showed a smooth mucosal surface more frequent in isolated IgG4-SC (75% vs. 7%, *p* = 0.006). Only one patient had two pathological findings characteristic of IgG4-SC. The sensitivity for diagnosing PHCC was 81% using two or more combined sampling methods. Conclusions: Pathological examinations have limitations in the differential diagnosis of isolated-IgG4-SC and PHCC, and a diagnostic strategy that combines multiple ERCP-related procedures, including IDUS and POCS, is recommended.

## 1. Introduction

Immunoglobin (Ig)G4-related sclerosing cholangitis (IgG4-SC) is currently recognized as a manifestation of the systemic disorder IgG4-related disease [1]. IgG4-SC is characterized by elevated serum IgG4 levels and is frequently associated with autoimmune pancreatitis (AIP) [2,3]. In contrast, IgG4-SC without AIP is called isolated IgG4-SC [4,5]. IgG4-SC is classified into four types based on the site of strictures due to cholangiography and associated diseases: type 1, localized strictures only in the distal common bile duct; type 2, strictures involving the intrahepatic bile ducts; type 3, localized strictures in both the hilar and distal common bile ducts; type 4, strictures only in the hilar duct [6]. Isolated IgG4-SC is extremely rare, accounting for approximately 4% of all IgG4-SC; however, 30–50% of isolated IgG4-SC cases exhibit type 4 cholangiographic classification [5,7,8].

When diagnosing isolated IgG4-SC, most of which exhibit type 4 cholangiograms, it is important to differentiate them from perihilar cholangiocarcinomas (PHCCs). However, differentiating between these two diseases is a major challenge, and there are many reports of isolated IgG4-SC misdiagnosed as PHCC and consequently surgically resected [9,10,11]. Although endoscopic retrograde cholangiopancreatography (ERCP) has risks such as post-ERCP pancreatitis [12], it is essential procedure for differential diagnosis of localized bile duct strictures. Pathological examination is effective in the differential diagnosis of benign or malignant bile duct strictures. However, the sensitivity of pathological examinations for the diagnosis of cholangiocarcinoma were reported at 48–74% [13,14,15,16,17]. In contrast, intraductal ultrasonography (IDUS) and peroral cholangioscopy (POCS), which are ERCP-related procedures, have been reported to be effective in differentiating IgG4-SC from cholangiocarcinoma [18,19,20,21,22,23,24]. There have been several reports on the clinical features of isolated IgG4-SC or the differential diagnosis of IgG4-SC and extrahepatic cholangiocarcinoma [4,5,7,8,18,19,20,21,22,23,24]; however, there have been no reports on the utility of ERCP-related procedures, including both IDUS and POSC, in the differential diagnosis of isolated IgG4-SC and PHCC.

This study aimed to clarify the utility of ERCP-related procedures, including IDUS, POCS, and pathological examination for the differential diagnosis of isolated IgG4-SC and PHCC.

## 2. Materials and Methods

### 2.1. Patients

**Figure 1:**
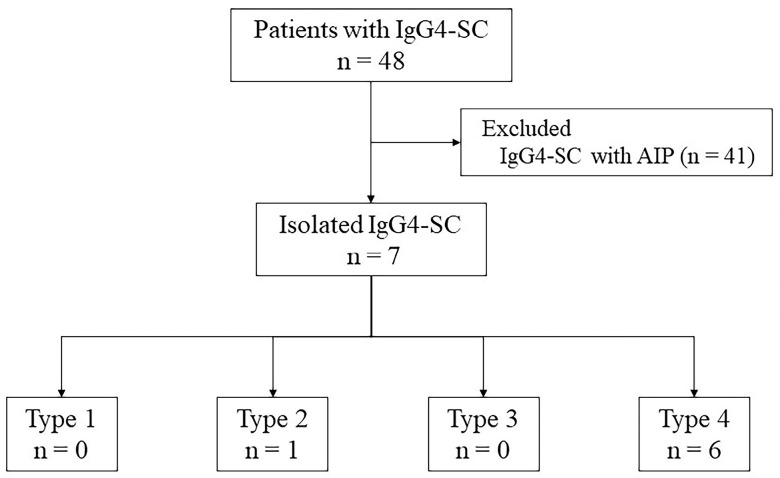
Breakdown of the patients diagnosed with IgG4-SC. Forty-one patients were diagnosed with IgG4-SC and AIP, and seven patients were diagnosed with isolated IgG4-SC. Among patients with isolated IgG4-SC, one patient had a type 2 biliary stricture and six patients had a type 4 biliary stricture. AIP, autoimmune pancreatitis; IgG4-SC, IgG4 related sclerosing cholangitis.

Patients with isolated IgG4-SC and infiltrating-type PHCC who underwent ERCP-related procedures at the Hiroshima University Hospital between January 2004 and December 2022 were enrolled. Overall, 48 patients were diagnosed with IgG4-SC. Of these patients, 41 had autoimmune pancreatitis, and seven were finally diagnosed with isolated IgG4-SC (Figure 1). Of the seven patients with isolated IgG4-SC, six showed cholangiographic classification type 4, and one showed type 2. All patients with PHCC underwent surgical resection and pathological confirmation. Infiltrating PHCC consist of macroscopically nodular and flat infiltrating types [25]. Isolated IgG4-SC was diagnosed based on the clinical diagnostic criteria for IgG4-SC [26]. IDUS and POCS were performed to differentiate between benign and malignant bile duct strictures or to diagnose the extent of the superficial spread of PHCC.

This study was conducted in accordance with the Declaration of Helsinki and approved by the ethics committee of Hiroshima University Hospital (approval number: E2023-0172). Written informed consent was obtained from all patients and their families before the ERCP was performed.

### 2.2. ERCP-Related Procedures

ERCP was performed using a video duodenoscope (JF-260V, TJF-240, or TJF-260V; Olympus Medical Systems, Tokyo, Japan), and IDUS was performed using an ultrasound probe UM-G20-29R (Olympus Medical Systems, Tokyo, Japan). All POCS procedures were performed using the conventional dual operator mother-baby technique. One of the three video cholangioscopes (CHF-B260, CHF-B290, and CHF-BP260; outer diameter of 3.4 mm, 3.3 mm, and 2.6 mm; working channel diameter of 1.2 mm, 1.3 mm, and 0.5 mm, respectively; Olympus Medical Systems, Tokyo, Japan) was used as a baby scope. CHF-B260 or CHF-BP260 were used until September 2020, and CHF-B290 was used after October 2020. For bile duct cannulation, a contrast cannula loaded with a 0.025-inch guidewire was used. In cases which conventional bile duct canulation was difficult, a pancreatic guidewire-assisted technique or pancreatic sphincterotomy were performed.

During the initial ERCP, cholangiography was performed, followed by IDUS. Bile duct brush cytology of the stricture site was performed, and an endoscopic retrograde nasobiliary drainage (ENBD) catheter was placed after endoscopic sphincterotomy. The cytology brushes used were RX Cytology Brush (Boston Scientific Corp., Marlborough, MA, USA). The ENBD catheters used were 4-Fr or 6-Fr ENBD catheter (Gadelius Medical, Tokyo, Japan) and 6-Fr. FleximaTM Nasobiliary Catheter (Boston Scientific Corp., Marlborough, MA, USA). On the day after the initial ERCP, a detailed evaluation of the bile duct was performed using cholangiography via an ENBD catheter. Bile juice cytology was performed multiple times during the initial ERCP or by using an ENBD catheter. In secondary ERCP, POCS was performed if necessary, and a forceps biopsy was performed. The biopsy forceps used were Radial JawTM 4 (Boston Scientific Corp., Marlborough, MA, USA). In patients with jaundice or acute cholangitis, a secondary ERCP was performed after improvement. All ERCP-related procedures were performed under conscious sedation with intravenous administration of midazolam alone or midazolam plus pentazocine.

### 2.3. Definitions

IDUS findings were evaluated based on the following previously reported criteria as follows [18,19]: (1) bile duct wall thickness, (2) symmetry of wall thickening at the stricture site of the bile duct (symmetry or asymmetry), (3) outer margin of the bile duct (smooth or irregular), (4) internal echo (homogeneous or heterogeneous), and (5) wall thickness outside the stricture site.

POCS findings were evaluated based on previously reported points as follows [20,21,22,23]: (1) properties of the mucosal surface of the bile duct, smooth or irregular (papillogranular); (2) existence of dilated vessels and their morphology (tortuosity, abrupt caliber alteration, or disruption); (3) easy bleeding. Easy bleeding was defined as spontaneous bleeding without contact with a guidewire or a cholangioscope. All IDUS and cholangioscopic images were stored as both video and still images and were evaluated by two endoscopists with over 10 years of experience (Y.I. and S.N.).

Bile and brush cytology were performed using conventional Papanicolaou staining, and cytological diagnoses were categorized into the following three groups: negative (normal or reactive process), atypical (atypical cells of unknown malignancy), and positive (strongly suspected or definite malignancy). The pathological diagnosis of IgG4-SC was confirmed based on the presence or absence of the following four findings according to the clinical diagnostic criteria [26]: (1) marked lymphoplasmacytic infiltration and fibrosis, (2) >10 IgG4-positive plasma cells per high-power microscopic field, (3) storiform fibrosis, and (4) obliterative phlebitis. The diagnosis of isolated IgG4-SC was based on clinical diagnostic criteria [26], and categorized as “definite”, “probable”, and “possible”.

### 2.4. Statistical Analysis

Statistical analyses were performed using the JMP software (Version Pro 17.0.0; SAS Institute Inc., Tokyo, Japan). Continuous variables were compared using the Wilcoxon rank-sum test, and categorical values were compared using the chi-square or Fisher’s exact tests. *p* values < 0.05 were considered statistically significant.

## 3. Results

### 3.1. Patient Characteristics of Isolated IgG4-SC

The clinical characteristics of the patients with isolated IgG4-SC are shown in Table 1. The median age was 59 years, and five patients were men and two were women. The median serum IgG4 level was 324 mg/dL, and elevated serum IgG4 levels (≥135 mg/dL) were observed in all seven patients. Only one patient had sclerosing dacryoadenitis/sialadenitis with the involvement of other organs. Six patients were treated with steroids, and one patient experienced spontaneous remission. According to the diagnostic criteria, one of the six patients had a “definite diagnosis”, three patients had “probable diagnosis”, and two patients had “possible diagnosis”.

### 3.2. Comparison of Clinical Factors between Hilar Type Isolated IgG4-SC and PHCC

Table 2 shows the comparison of clinical factors between the 6 patients with isolated IgG4-SC and 65 patients with PHCC. The median age was significantly higher in the PHCC group (62 vs. 72 years, *p* = 0.007). Serum IgG (1953 mg/dL vs. 1288 mg/dL, *p* = 0.006) and IgG4 (265 mg/dL vs. 43 mg/dL, *p* < 0.001) levels were significantly higher in the IgG4-SC group. One patient (4%) in the PHCC group also had a serum IgG4 level ≥ 135 mg/dL (199 mg/dL), and the absence of concomitant IgG4-related diseases was assessed by computed tomography. There were no significant differences in age, sex, total bilirubin levels, or liver enzyme levels between the two groups.

### 3.3. IDUS and POCS Findings

The IDUS and POCS findings of isolated IgG4-SC and PHCC are shown in Table 3. There was no significant difference in bile duct thickness (2.9 mm vs. 3.2 mm, *p* = 0.806). Symmetrical bile duct wall thickness (40% vs. 5%, *p* = 0.040) and homogenous internal echo (80% vs. 5%, *p* < 0.001) at the stricture site and smooth outer margins of the bile duct (80% vs. 6%, *p* < 0.001) were more frequently observed in the IgG4-SC group. Wall thickness outside the stricture site was also frequently observed in the IgG4-SC group (100% vs. 0%, *p* < 0.001).

Four patients in the isolated IgG4-SC group and 30 in the PHCC group underwent POCS. Smooth mucosal surfaces were observed significantly more frequently in the IgG4-SC group (75% vs. 7%, *p* = 0.006). Dilated vessels were observed in both groups (50% vs. 80%, *p* = 0.229). Although the differences were not significant, tortuosity (25% vs. 60%, *p* = 0.229), abrupt caliber alternation (25% vs. 67%, *p* = 0.274), and vessel disruption (0% vs. 43%, *p* = 0.145) were observed more frequently in the PHCC group. Easy bleeding was also observed more frequently in the PHCC group (0% vs. 53%, *p* = 0.105).

In this study, the significance level was set at 0.05. For IDUS findings, statistical powers of symmetry of wall thickening, internal echo, outer margin and wall thickening at the non-stricture site were 0.35, 0.98, 0.98 and 0.99, respectively. For POCS findings, statistical powers of mucosal surface, dilated vessels, tortuosity, abrupt caliber alteration, disruption and easy bleeding were 0.87, 0.21, 0.32, 0.44, 0.99 and 0.99, respectively.

### 3.4. Pathological Diagnosis

The pathological findings of the forceps biopsy in patients with isolated IgG4-SC are shown in Table 4. Lymphocytic and plasmacyte infiltration and fibrosis were observed in four of six patients, and IgG4-positive plasma cells > 10/HPF were observed in one patient. Storiform fibrosis and obliterative phlebitis were not observed in any of the six patients, and no patients were diagnosed pathologically alone.

The diagnostic yields of 65 patients with PHCC are shown in Table 5. Bile cytology was performed multiple times, with a median of seven times in the isolated IgG4-SC group and three times in the PHCC group. The sensitivities of bile juice cytology, bile duct brush cytology, and bile duct biopsy were 39%, 44%, and 58%, respectively. Specificity was 100% for all pathological examinations.

### 3.5. Comparison of Diagnostic Yields of ERCP-Related Procedures

The diagnostic yields of IDUS, POCS, and pathological diagnoses are presented in Table 5. Of these three procedures, IDUS showed the best diagnostic performance. When two or more findings suggestive of malignancy were observed, the sensitivity, specificity, and accuracy were 97%, 80%, and 96%, respectively. In POCS, mucosal irregularity showed a relatively high diagnostic performance, with a sensitivity of 93% and an accuracy of 91%. When two or more findings of an irregular mucosal surface, dilated vessels, or easy bleeding were observed, the sensitivity, specificity, and accuracy were 77%, 75%, and 76%, respectively. In histopathological diagnosis, the sensitivity was 81%, specificity was 100%, and accuracy was 83% when two or more bile cytology, brush cytology, or forceps biopsy sampling methods were performed.

**Figure 2:**
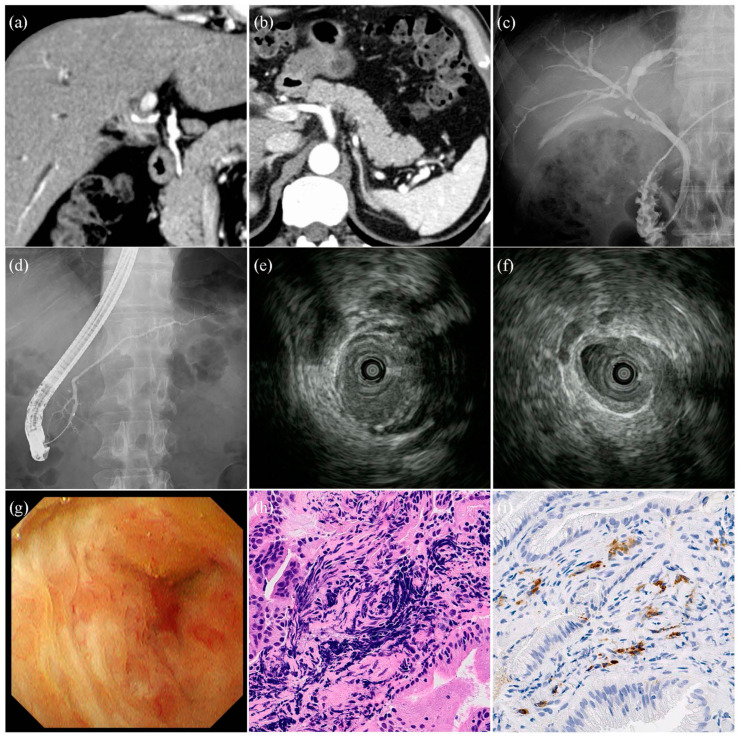
Images of the patients with isolated IgG4-related sclerosing cholangitis. (**a**,**b**) Contrast-enhanced computed tomography images. Bile duct stricture and wall thickness in the perihilar region of the bile duct. The pancreas is normal, without findings of autoimmune pancreatitis. (**c**) Cholangiographic image. Stricture in the perihilar region of the bile duct. (**d**) Pancreatographic image. Normal pancreatic duct. (**e**) Intraductal ultrasonographic image of the stricture site. Wall thickness of bile duct was asymmetrical, internal echo was homogeneous, and lateral margin of bile duct was smooth. (**f**) Intraductal ultrasonographic image outside the stricture site. Symmetrical wall thickness of bile duct. (**g**) Cholangioscopic image in the stricture site. Mucosal surface was regular, and irregular vessels were absent. (**h**) Hematoxylin-Eosin stain (original magnification: ×800). Lymphocytic and plasmacyte infiltration and fibrosis detected by forceps biopsy of the stricture site. (**i**) IgG4-positive plasma cells detected by forceps biopsy of the stricture site (original magnification: ×800).

A case of isolated IgG4-SC is shown in Figure 2. A 51-year-old man presented with jaundice. Labora tory investigations revealed a serum IgG4 level of 630 mg/dL. Contrast-enhanced computed tomography (CE-CT) revealed bile duct thickening in the perihilar region. IDUS showed that the thickening of the bile duct wall at the stricture site was asymmetric; however, the internal echo was homogeneous, and the outer margin of the bile duct wall was smooth. Thickening of the bile duct wall outside the stricture site was also observed. In the POCS group, the mucosal surface of the strictures was smooth. Dilated vessels were observed; however, no tortuosity or disruption of the vessels was observed. In the pathological diagnosis, lymphocytic and plasmacyte infiltration and IgG4 positive plasma cells were observed; however, there were no findings suggestive of malignancy.

## 4. Discussion

The differential diagnosis between isolated IgG4-SC and PHCC is not easy. There were three reports about surgical resected isolated IgG4-SC [9,10,11]. In these reports, three of five patients were diagnosed as PHCC only by CT or magnetic resonance cholangiopancreatography (MRCP). Other two patients were received ERCP-related procedures, but surgically resection was performed because of the possibility of PHCC could not rule out. It may be due to the similarity of the two diseases and the fact that useful modality for differential diagnosis has not been clarified. So, we performed this study.

In this study, 86% (6/7) of patients with isolated IgG4-SC presented with cholangiographic classification type 4 hilar bile duct strictures. Many patients present with type 4 bile duct findings [7,8], and isolated IgG4-SC may be characterized by the main location of the lesion in the hilar bile duct. In contrast, Nakazawa et al. [27] reported several cases of type 1 isolated IgG4-SC presenting with a focal stricture of the intrapancreatic bile duct. In this study, the remaining patients had type 2 IgG4-SC, and none had type 1 IgG4-SC. Type 1 IgG4-SC is often associated with AIP [7,8]; therefore, when diagnosing type 1 isolated IgG4-SC, its occurrence after AIP improvement should be considered. To clarify the cholagiographic classification of isolated IgG4-SC, future studies warrant the inclusion of a large number of cases.

Elevated serum IgG4 levels are an important finding with excellent sensitivity and specificity for IgG4-SC diagnosis [28]. Under the current diagnostic criteria [26], elevated serum IgG4 levels are necessary for a definitive diagnosis in patients without AIP. A large Japanese cohort study reported no difference in the frequency of elevated serum IgG4 levels (≥135 mg/dL) between patients with IgG4-SC with and without AIP. In the present study, all seven patients with isolated IgG4-SC showed high serum IgG4 levels. In contrast, several studies have reported that isolated IgG4-SC cases are often not accompanied by elevated serum IgG4 levels [4,27], and many of these cases were diagnosed as cholangiocarcinoma and underwent surgical resection. Although it is difficult to differentiate isolated IgG4-SC without elevated IgG4 levels from cholangiocarcinoma, comprehensive use of ERCP-related procedures, including pathological examinations, IDUS, and POCS, may lead to an accurate diagnosis.

Pathological diagnosis is one of the most reliable methods for the differential diagnosis between IgG4-SC and PHCC. However, it is difficult to distinguish between these two diseases based on pathological diagnosis alone. In particular, the diagnostic yield of histopathological diagnosis in patients with IgG4-SC is poor, and no patient in this study was diagnosed with IgG4-SC based on histopathology alone. Nakazawa et al. reported that among five patients with isolated intrapancreatic IgG4-SC, only one exhibited characteristic findings of IgG4-SC on forceps biopsy [27]. Takagi et al. reported two patients with characteristic findings of IgG4-SC on forceps biopsy among nine patients with isolated proximal-type IgG4-SC [29]. This problem occurs not only in cases of isolated IgG4-SC but also in those of whole IgG4-SC [20,24,28]. One reason for the difficulty in diagnosing IgG4-SC histopathologically is the location of inflammation. The fibroinflammatory involvement is observed mainly in the stroma of the bile duct wall in IgG4-SC, and the bile duct epithelium is often histologically normal [27]. Therefore, it may be necessary to develop instruments capable of sampling the stroma of the bile duct, such as biopsy forceps with larger cups, to improve the pathological diagnostic performance of IgG4-SC. ERCP-related procedures, such as IDUS and POCS, are important diagnostic methods because of the difficulty in the pathological diagnosis of isolated IgG4-SC.

IDUS or POCS have been used to differentiate IgG4-SC from extrahepatic cholangiocarcinoma (ECC). IDUS findings such as asymmetrical wall thickening, heterogeneous internal echo, or irregular lateral margins of the bile duct have been reported to be effective in diagnosing ECC [18], and wall thickening outside the stricture site has been reported as a characteristic finding of IgG4-SC [19]. In our study, these findings were beneficial in diagnosing IgG4-SC. Ishii et al. reported that the diagnosis of ECC had 96% (51/53) sensitivity and 89% (17/19) specificity when the POCS findings of malignant strictures were defined as an irregular mucosal surface, presence of dilated vessels with abrupt caliber alteration and disruption, or easy bleeding [23]. In our study, both the sensitivity and specificity were lower than those in previous studies when examined under similar definitions. In isolated IgG4-SC, IDUS was more useful than POCS for the differential diagnosis. However, there were two patients with PHCC who could not be diagnosed by IDUS or histopathological examination and were diagnosed with POCS. For a more accurate diagnosis, multiple examinations are required, including POSC and IDUS, in patients with bile duct strictures that are difficult to differentiate.

Recently, the variety of devices available for POCS has increased because of the development of new equipment. The SpyGlass DS Direct Visualization System (Boston Scientific Corporation, Natick, MA, USA) has been presented as a novel approach for treating biliary diseases. While most of these approaches are related to the treatment of bile duct stones, Minami et al. reported on the diagnosis of biliary tract diseases [30]. In our institution, conventional video mother-baby-type cholangioscopes are used because their image quality is higher than that of the SpyGlass DS. The Narrow Band Imaging (NBI) mode is also effective in observing vessel patterns but is limited to conventional cholangioscopes. In contrast, the SpyGlass DS allows direct observation of the spot where a forceps biopsy is needed. In fact, the diagnostic yields in Minami’s report [30] were better than those in previous reports [13,14,15,16,17] and in our results using conventional cholangioscopes. However, the SpyGlass DS has disadvantages such as being disposable and expensive. Further studies are required to determine the appropriate equipment.

## 5. Limitations

This study has some limitations. First, it was a retrospective study with a relatively small number of patients. The small number of patients with isolated IgG4-SC may make this study statistically unreliable. Isolated IgG4-SC is a rare disease, and multicenter studies with a large number of patients are required. Second, no patient with isolated IgG4-SC underwent surgical resection; therefore, histological findings could not be effectively compared with IDUS or POCS findings in patients with isolated IgG4-SC. However, it is difficult to obtain isolated IgG4-SC histopathological findings. This is because there are only a few patients with isolated IgG4-SC, and fewer cases are surgically resected owing to advances in clinical practice guidelines [31] and diagnostic criteria [26]

## 6. Conclusions

Isolated IgG4-SC is a rare disease, but it is important to differentiate from PHCC because most of them occur hilar region bile duct stricture. Although diagnosis of isolated IgG4-SC is not easy, in addition to pathological examinations, detailed observation of biliary strictures using IDUS and POCS may help differentiate isolated IgG4-SC from PHCC.

## Figures and Tables

**Table 1:** Clinical characteristics of the patients with isolated IgG4-SC.

Case	Age (Years)	Sex	Serum IgG4 (mg/dL)	Cholangiographic Classification	Other Organ Involvement	Treatment	Diagnosis *
1	67	Female	206	Type 4	None	Steroid	Possible
2	68	Male	194	Type 4	None	Spontaneous remission	Possible
3	70	Female	324	Type 4	None	Steroid	Probable
4	51	Male	630	Type 4	None	Steroid	Definite
5	57	Male	179	Type 4	None	Steroid	Probable
6	45	Male	530	Type 4	None	Steroid	Probable
7	59	Male	2580	Type 2	Dacryoadenitis/ sialadenitis	Steroid	Definite

* Diagnosis was made according to the clinical diagnostic criteria for IgG4-SC 2020 [26]. IgG4-SC, IgG4-related sclerosing cholangitis.

**Table 2:** Comparison of clinical factors between hilar type isolated IgG4-SC and PHCC.

Parameters	Isolated IgG4-SC (*n* = 6)	PHCC (*n* = 65)	*p* Value
Age (years)	62 (50–69)	72 (67–77)	0.007
Sex (male/female)	4/2	50/15	0.625
Serological findings			
T-Bil (mg/dL)	2.0 (1.1–12.2)	1.6 (1.0–8.0)	0.934
AST (U/L)	46 (21–154)	94 (44–159)	0.189
ALT (U/L)	58 (20–361)	125 (52–249)	0.380
ALP (U/L)	482 (413–1071)	891 (548–1536)	0.139
CEA (ng/mL)	2.7 (1.8–3.9)	2.6 (2.3–4.6)	0.463
CA19-9 (U/mL)	19 (5–62)	69 (14–238)	0.114
IgG (mg/dL)	1953 (1298–2093)	1288 (1075–1501)	0.006
IgG4 (mg/dL)	265 (190–555)	42.9 (24–81)	<0.001
IgG4 ≥ 135 mg/dL	6/6 (100%)	1/26 (4%)	<0.001

Data are expressed as median (interquartile range). IgG4-SC, IgG4 related sclerosing cholangitis; PHCC, perihilar cholangiocarcinoma; T-Bil, total bilirubin; AST, aspartate aminotransferase; ALT, alanine aminotransferase; ALP, alkaline phosphatase; CEA, carcinoembryonic antigen; CA19-9, carbohydrate antigen 19-9; IgG, immunoglobulin G Reference values of blood analysis; T-Bil, 0.4–0.5 mg/dL; AST, 13–30 U/mL; ALT, 10–42 U/mL; ALP, 106–322 U/mL; CEA, 0–5 ng/mL; CA19–9, 0–37 U/mL, IgG, 861–1747 mg/dL; IgG4, 11–120.9 mg/dL.

**Table 3:** Comparison of IDUS and POCS findings between hilar type isolated IgG4-SC and PHCC.

	Isolated IgG4-SC (*n* = 5)	PHCC (*n* = 65)	*p* Value
**IDUS findings**			
Thickness of bile duct wall (mm)	2.9 (2.6–5.6)	3.2 (1.2–11)	0.806
Wall properties at the stricture site			
Symmetry of wall thickening (symmetric/asymmetric)	2 (40)/3 (60)	3 (5)/60 (95)	0.040
Internal echo (homogeneous/heterogeneous)	4 (80)/1 (20)	3 (5)/60 (95)	<0.001
Outer margin (smooth/irregular)	4 (80)/1 (20)	4 (6)/59 (94)	<0.001
Wall thickening at the non-stricture site (present/absent)	5 (100)/0 (0)	0 (0)/63 (100)	<0.001
**POCS findings**			
Mucosal surface (smooth/irregular)	3 (75)/1 (25)	2 (7)/28 (93)	0.006
Dilated vessels (present/absent)	2 (50)/2 (50)	24 (80)/6 (20)	0.229
Tortuosity (present/absent)	1 (25)/3 (75)	18 (60)/12 (40)	0.299
Abrupt caliber alteration (present/absent)	1 (25)/3 (75)	20 (67)/10 (33)	0.274
Disruption (present/absent)	0 (0)/4 (100)	13 (43)/17 (57)	0.145
Easy bleeding (present/absent)	0 (0)/4 (100)	16 (53)/14 (47)	0.105

Data are expressed as median (interquartile range) and number (percentage). IgG4-SC, IgG4-related sclerosing cholangitis; PHCC, perihilar cholangiocarcinoma; IDUS, intraductal ultrasonography; POCS, peroral cholangioscopy.

**Table 4:** Pathological findings of forceps biopsy in patients with hilar type isolated IgG4-SC.

	Case 1	Case 2	Case 3	Case 4	Case 5	Case 6
Lymphocytic and plasmacyte infiltration and fibrosis	Absent	Present	Present	Present	Absent	Present
IgG4-positive plasma cells > 10/HPF	Absent	Absent	Absent	Present	Absent	Absent
Storiform fibrosis	Absent	Absent	Absent	Absent	Absent	Absent
Obliterative phlebitis	Absent	Absent	Absent	Absent	Absent	Absent
Number of findings	0	1	1	2	0	1

IgG4-SC, IgG4-related sclerosing cholangitis; HPF, high-power field.

**Table 5:** Diagnostic yields of ERCP-related procedures in differentiating between PHCC and isolated IgG4-SC.

	Sensitivity	Specificity	PPV	NPV	Accuracy
IDUS					
Asymmetric wall thickening	95 (60/63)	40 (2/5)	95 (60/63)	40 (2/5)	91 (62/68)
Heterogeneous internal echo	95 (60/63)	80 (4/5)	98 (60/61)	57 (4/7)	94 (64/68)
Irregular outer margin	94 (59/63)	80 (4/5)	98 (59/60)	50 (4/8)	93 (63/68)
Wall thickening at the non-stricture site	100 (63/63)	100 (5/5)	100 (63/63)	100 (5/5)	100 (68/68)
Two or more above findings	97 (61/63)	80 (4/5)	98 (61/62)	67 (4/6)	96 (65/68)
POCS					
Irregular mucosal surface	93 (28/30)	75 (3/4)	97 (28/29)	60 (3/5)	91 (31/34)
Dilated vessels	67 (20/30)	75 (3/4)	95 (20/21)	23 (3/13)	68 (23/34)
Easily bleeding	53 (16/30)	100 (4/4)	100 (16/16)	22 (4/18)	59 (20/34)
Two or more above findings	77 (23/30)	75 (3/4)	96 (23/24)	30 (3/10)	76 (26/34)
Pathological examinations					
Bile cytology	39 (25/64)	100 (5/5)	100 (25/25)	11 (5/44)	44 (30/69)
Brush cytology	44 (27/62)	100 (5/5)	100 (27/27)	13 (5/40)	48 (32/67)
Forceps biopsy	59 (38/65)	100 (6/6)	100 (38/38)	18 (6/33)	62 (44/71)
Two or more above sampling methods	81 (53/65)	100 (5/5)	100 (53/53)	29 (5/17)	83 (58/70)

Data are expressed as percentage (number). ERCP, endoscopic retrograde cholangiopancreatography; PHCC, perihilar cholangiocarcinoma; IgG4-SC, IgG4-related sclerosing cholangitis; PPV, positive predictive value; NPV, negative predictive value; IDUS, intraductal ultrasonography; POCS, peroral cholangiography.

## Data Availability

The data presented in this study are available on request from the corresponding author. The data are not publicly available due to privacy issues.
